# Coupling Effects of Nitrogen and Irrigation Levels on Growth Attributes, Nitrogen Use Efficiency, and Economics of Cotton

**DOI:** 10.3389/fpls.2022.890181

**Published:** 2022-05-16

**Authors:** Rakesh Kumar, Narendra Kumar Pareek, Uttam Kumar, Talha Javed, Asma A. Al-Huqail, Vijay Singh Rathore, Vinay Nangia, Ashok Choudhary, Gangadhar Nanda, Hayssam M. Ali, Manzer H. Siddiqui, Ahmed F. Youesf, Arkadiusz Telesiński, Hazem M. Kalaji

**Affiliations:** ^1^College of Agriculture, Swami Keshwanand Rajasthan Agricultural University (SKRAU), Bikaner, India; ^2^College of Plant Protection, Fujian Agriculture and Forestry University, Fuzhou, China; ^3^College of Agriculture, Fujian Agriculture and Forestry University, Fuzhou, China; ^4^Department of Agronomy, University of Agriculture Faisalabad, Faisalabad, Pakistan; ^5^Department of Botany and Microbiology, College of Science, King Saud University, Riyadh, Saudi Arabia; ^6^Regional Research Station, CAZRI, Bikaner, India; ^7^International Center for Agricultural Research in the Dry Areas (ICARDA), Aleppo, Syria; ^8^College of Agriculture, G. B. Pant University of Agriculture and Technology, Pantnagar, India; ^9^Animal Production Research Institute, Dr. Rajendra Prasad Central Agricultural University, Pusa, India; ^10^Department of Horticulture, College of Agriculture, University of Al-Azhar, Asyut, Egypt; ^11^Department of Bioengineering, West Pomeranian University of Technology, Szczecin, Poland; ^12^Department of Plant Physiology, Institute of Biology, Warsaw University of Life Sciences-SGGW, Warsaw, Poland; ^13^Institute of Technology and Life Sciences - National Research Institute, Falenty, Poland

**Keywords:** irrigation, sustainability, nitrogen implantation dosage, cotton, nitrogen use efficiency

## Abstract

Nitrogen (N) fertilization plays a pivotal role in physiomorphological attributes and yield formation of field-grown cotton (*Gossypium hirsutum* L.), but little is known of its interaction with irrigation levels. Therefore, this study was conducted with an objective of evaluating the impact of irrigation and nitrogen levels on growth attributes and nitrogen use efficiency of *Bt* cotton (*Gossypium* spp.) in the hot arid region. The experiment consisted of a factorial arrangement of three irrigation levels (200, 400, and 600 mm) and four nitrogen rates (0, 75, 150, and 225 kg ha^–1^) in a split-plot design with three replications. Nitrogen fertilization and irrigation levels influenced cotton growth attributes and yield. The highest leaf area index, dry matter accumulation, crop growth rate, and relative growth rate were achieved at 225 kg N ha^–1^ and irrigation level 600 mm as compared to other experimental treatments. Similarly, nitrogen uptake and content by seed, lint, and stalk and total nitrogen uptake recorded maximum at 225 kg N ha^–1^ and irrigation level 600 mm. Interestingly, the treatment of 600 mm of irrigation and 150 kg N ha^–1^ displayed significant increase in nitrogen use efficiency indices such as agronomic efficiency of nitrogen (AEN) and recovery efficiency of nitrogen (REN), while partial factor productivity of nitrogen (PFPN) and internal nitrogen use efficiency (iNUE) were significantly higher with application of 600 mm of irrigation and nitrogen application rate of 75 kg ha^–1^. Application of 600 mm of irrigation along with 225 kg N ha^–1^ resulted in significant increase in gross return, net return, and B:C ratio than any other treatment combinations. So, application of 600 mm of irrigation along with 225 kg N ha^–1^ could be recommended for achieving higher growth and yield, as well as profitability of *Bt* cotton under hot arid region and similar agroecologies.

## Introduction

Cotton (*Gossypium* spp.) is India’s leading fiber cash crop, accounting for 85% of the textile industry’s raw materials. India is the world’s leading cotton cultivator and producer, trailing only China. Cotton is grown on 129.57 lac ha in India, with a production and productivity of 371.0 lac bales and 486.76 kg ha^–1^, respectively, which is lower than the global average (Mahadevappa et al., 2018). The poor growth and productivity of *Bt* cotton can be attributed to a number of reasons, including untimely rain, late and early onset of monsoon, improper water management, improper sowing time, and imbalanced use of fertilizers (Ramasundaram and Gajbhiye, 2001). Among these factors, availability of water is the major factor as more than 50% of cotton is growing under rainfed condition, where low and erratic rainfalls affect the cotton growth and ultimately affect the productivity. Cotton is a semi-xerophyte and forced annual crop. Its vegetative growth and duration are linearly related to water supply and fertilization over a wide input range. However, harvest index is higher at lower water supplies. Thus, a greater proportion of total biomass is proportioned to reproductive structures as water stress is increased. This underlying principle entails that cotton needs only lifesaving irrigation to circumvent severe stress at the critical boll development stage (Wang et al., 2018). Similarly, inherent poor soil fertility, particularly soils low in N content and micronutrients, is a major factor responsible for the low productivity of cotton in arid and semi-arid environment (Wang et al., 2018; Shah et al., 2021a). Limited soil water availability during important growth periods such as flowering and boll production can result in fewer bolls, boll shedding, and underdeveloped bolls. As a result, irrigation is the main factor in ensuring *Bt* cotton’s long-term growth and productivity because efficient water use through a well-managed irrigation plan is critical for crop output, especially in light of deteriorating land and water availability per capita (Mahmood et al., 2021).

Nitrogen (N) plays a prominent role in the plant metabolic system (Shah et al., 2017, 2021b). Nitrogen is a principal component of many organic compounds (proteins, nucleic acids, alkaloids, and enzymes) and is also associated with energy transfer molecules such as ADP and ATP. It consists of 16% of total protein biomass present in plants (Ahmad et al., 2022). N is also found in nucleic acids (DNA and RNA), which play an important role in plant genetics and heredity. It is also a component of chlorophyll, which serves as a photosynthesis factory. N has also major role in photosynthetic processes, leaf area production, leaf area duration, as well as net assimilation rate that are directly related to yield enhancement (Leghari et al., 2016). More than half of the world’s population is fed by crops cultivated using synthetic nitrogen (N) fertilizers, which were made feasible by the advent of the Haber–Bosch process in the early twentieth century, which converts atmospheric nitrogen gas (N_2_) to active forms of nitrogen. Total global consumption of N fertilizer was 112.5 million tons in 2015, 118.2 million tons in 2019, and likely to reach 7.9–10.5 billion tons by 2050 (Zhang X. et al., 2015). The demand of huge amount of N fertilizers is a major concern for farmers as it increases the cost of cultivation and also degrades the inherent fertility of soil. Consequently, the use of excess N fertilizers causes the problem of N pollution, which is now considered as new threat for environmental sustainability (Kanter et al., 2020). It has been documented that the nitrogen use efficiency (NUE) value is decreased with the application of N fertilizers. For instance, in developed countries, NUE increased because of adaption of best agronomical and fertilizer management practices and reduced in developing countries such as India as the use of N fertilizer dramatically increased (Heffer and Prud’homme, 2016). It was reported that the world average NUE was nearly 47 and 42% in 2009 and 2010, respectively, and the major reason behind the NUE decreasing is adoption of agronomical practices (nutrient management and varieties) associated with low NUE (Yousaf et al., 2014, 2016, 2021; Zhang Y. et al., 2015).

Different levels of water and fertilizers have significant combined effects on yield, crop growth, and water productivity (Javed et al., 2022). The seed cotton yield has been shown to increase with an increase in N from 0 to 200 kg ha^–1^ under a high water supply. However, the seed cotton yield was found to first increase and then decrease with a lower water supply (Singh et al., 2010). Under fertigation, the water-use efficiency (WUE) decreased with a reduction in the amount of applied N and dense paired sowing resulted in higher seed cotton yield and WUE than normal sowing (Shah et al., 2021a). Cotton plants begin to take more nutrients on the 30th day after emergence, shortly after the start of flower budding, and achieve their daily maximum absorption during the flowering phase, 60–90 days following germination. Even in modern cotton production systems, where the crop is grown as an annual, there are phenological periods wherein vegetative growth and fruit development occur simultaneously. This behavior presents a problem for crop management in terms of maximizing nutrient uptake and ensuring an appropriate supply of water to allow the vegetative and reproductive parts of the crop to grow at the same time (Constable and Bange, 2015). As a result, this study was carried out to see how water and nitrogen levels affected cotton in Rajasthan’s desert climate. The purpose of this study was also to determine how different quantities of water and nitrogen effect cotton development, nutrient absorption, and economics.

## Materials and Methods

### Experimental Site

The experiment was conducted at fields of ARSS, Hanumangarh, which is a part of the Swami Keshwanand Rajasthan Agricultural University, Bikaner. It is located between 074°20′34′′E longitude and 28°37′62′′N latitude. This area comes under IB agroclimatic zone of Rajasthan and the Trans-Gangetic Plains region (VI) of India.

### Climatic Data and Soil Characteristics

Arid type of climate prevails in this area, with temperature extremes in both the summer and winter. The hottest months are May and June and the coldest month is January. During the summer, dust storms are very common in this area. The tract’s typical annual rainfall is around 300–500 mm, with the most of it falling between July and September. Fogs can be found in the area throughout the months of December and January.

**FIGURE 1:**
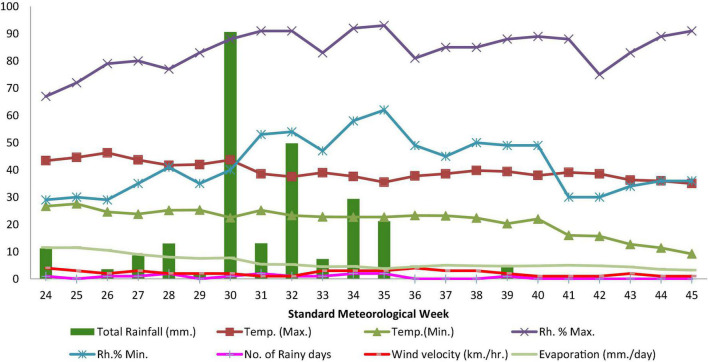
Mean weekly meteorological data during the crop season (Kharif, 2016).

Because weather has an impact on crop development, production, and quality, average weekly meteorological factors for the growing crops season should be taken into account. As there is no meteorological observatory established in Hanumangarh district of Rajasthan, hence weekly weather data obtained from nearby district [recorded at meteorological observatory of the Indian Meteorological Department (IMD) located at Sri Ganganagar] are shown in Table 1 and Figure 1.

**TABLE 1:** Mean meteorological data of experimental site during the crop season (Kharif, 2016).

Parameters	Values
Maximum temperature (°C)	30.5–46.3
Minimum temperature (°C)	8.6–27.6
Maximum relative humidity (%)	67–92
Minimum relative humidity (%)	29–62
Total rainfall (mm)	253.8
Total no. of rainy days	15
Evaporation (mm day^–1^)	3.3–11.5

The soil samples were randomly taken from 0 to 30 cm depth from ten different sites covering the experimental area in the season before planting for cotton to determine the physiochemical parameters. The values for various soil physiochemical properties are given in Table 2.

**TABLE 2:** Results of soil physicochemical properties in the study area.

Parameters	Values
pH	7.62
EC (dSm^–1^)	0.23
Bulk density (Mg m^–3^)	1.43
Organic carbon (%)	0.19
Available nitrogen (kg ha^–1^)	196.57
Available phosphorus (kg ha^–1^)	33.65
Available potassium (kg ha^–1^)	378.12
Soil texture	Silty clay

According to the results of the physiochemical analysis, the soils under examination had a silty clay texture and a slightly alkaline nature. Organic carbon levels were low, available nitrogen levels were low, available phosphorus levels were medium, and available potassium levels were high.

### Crop Husbandry and Experimental Treatments

The experiment comprises with 12 factorial combination with three levels of irrigation [200 (I_200_), 400 (I_400_), and 600 mm (I_600_)] in main plot and four levels of nitrogen [0 (N_0_), 75 (N_75_), 150 (N_150_), and 225 kg N ha^–1^ (N_225_)] in subplots. A uniform presowing irrigation of 60 mm was applied to all the plots. The measured quantity of irrigation to each plot was conveyed via a 2-inch polyvinyl chloride (PVC) pipe fitted with water flow meter (Kranti). The plots receiving 200, 400, and 600 mm irrigation were irrigated at 4 (30, 60, 90, and 120 days), 6 (30, 45, 60, 75, 90, and 120 days), and 8 (30, 45, 60, 75, 90, 105, 120, and 135 days) times, respectively. The rate of application of water was 50 mm each irrigation in case of I200 treatment. In I400 treatments, the rate of application of water was 50 mm for first irrigation and 70 mm for rest of five irrigations. In case of I600 treatment, the first irrigation was of 50 mm, second irrigation was of 70 mm, and rest of six irrigations was of 80 mm. In three splits, nitrogen was given in the form of urea as basal, at 1st irrigation on 30 days, and the remaining at the time of bud formation on 90 days, according to the subplot treatment. The treatments for the subplots were randomized with the help of Fisher’s random number table (Fisher, 1992). The seeds were sown @ 1.8 kg ha^–1^ of cotton in lines spaced as per treatments and sowing was done on 16 June 2016.

### Leaf Area Index

In cotton, leaf area analyzer (Model LI-3100, United States) was used for leaf area measurement of randomly chosen plants at each stage. Leaves were taken from the plants after chopping from ground and packed in a polythene bag. The area of the leaves was measured as soon as they were brought to the laboratory. The following formula was used to calculate the leaf area index (LAI):


$$\text{LAI}=\frac{\text{Total}\,\text{leaf}\,\text{area}\,\text{plant}\,\left({\text{cm}}^{2}\right)}{\text{Ground}\,\text{area}\,\text{covered}\,\text{by}\,\text{the}\,\text{plant}\,\left({\text{cm}}^{2}\right)}$$

### Dry Matter Accumulation

One plant was chosen at random from each plot and carefully uprooted to estimate dry matter accumulation (DMA) at each stage. Plant samples were washed and roots of these plants were taken. The plants were dried in sunlight in paper bags for 2–3 days before being dried at 70°C for 24 h to get a constant weight. After complete drying, weight of plant samples was taken. It was then averaged to express as dry matter accumulation in gram per plant.

### Crop Growth Rate and Relative Growth Rate

The mean crop growth rate (CGR) was calculated as suggested by Hussain et al. (2018) from the periodical dry matter accumulation recorded and expressed in g m^–2^ day^–1^.


$$\text{CGR}=\frac{{\text{W}}_{2}-{\text{W}}_{1}}{\left({\text{t}}_{2}-{\text{t}}_{1}\right)\times \text{A}}$$

Where, W_2_ and W_1_ are the total dry weight of the plant (g) at time t_2_ and t_1_, respectively, and A is the ground area occupied by the plant under W_1_/W_2_ in m^2^.

The mean relative growth rate (RGR) of both the crops is calculated from dry weight measurement at t_1_ and t_2_ suggested by Blackman (1919).


$$\text{RGR}=\frac{{\text{Log}}_{\text{e}}\cdot {\text{W}}_{2}-{\text{Log}}_{\text{e}}\cdot {\text{W}}_{1}}{{\text{t}}_{2}-{\text{t}}_{1}}$$

Where, W_2_ and W_1_ are the total dry weight of the plant (g) at time t_2_ and t_1_ and it is expressed in mg g^–1^ day^–1^.

### Number of Branches per Plant

It was calculated by averaging the number of branches per plant recorded at harvest from five tagged plants within every plot.

### Nitrogen Use Efficiency Indices

The different NUE indices such as agronomic efficiency of nitrogen (AEN), recovery efficiency of nitrogen (REN), physiological efficiency of nitrogen (PEN), partial factor productivity of nitrogen (PFPN), and internal nitrogen use efficiency (iNUE) were worked (Peng et al., 2006; Meena A. K. et al., 2018; Ballester et al., 2021; Singh et al., 2021) as follows:


$$\begin{array}{c} \text{AEN}\left[\mathrm{kg}\mathrm{seed}\mathrm{cotton}\left(\mathrm{kg}N{\mathrm{ha}}^{-1}\right)\right]=\left(\text{SCYF}-\text{SCYC}\right)/\\ \text{AFN}\,\left(\text{TNUF}-\text{TNUC}\right) \end{array}$$

REN(%)=(TNUF-TNUC)× 100/AFN

$$\begin{array}{c} \text{PEN}\left[\mathrm{kg}\mathrm{seed}\mathrm{cotton}\left(\mathrm{kg}{\mathrm{TNU}}^{-1}\right)\right]=\left(\text{SCYF}-\text{SCYC}\right)/\\ \left(\text{TNUF}-\text{TNUC}\right) \end{array}$$

$$\text{PFPN}\,\left(\mathrm{kg}\,N^{-1}\right)=\text{SCYF}/\text{AFN}$$

iNUE=SCY/TNU

Where, SCYF denotes the fertilized plot’s seed cotton yield (kg ha^–1^), SCYC stands for the control plot’s seed cotton yield (kg ha^–1^), AFN is the amount of applied fertilizer N (kg ha^–1^), TNUF is total nitrogen uptake by the cotton crop from the control plot (kg ha^–1^), TNU stands for total nitrogen uptake by cotton crops, and SCY represents the seed cotton yield (kg ha^–1^). TNUC stands for total nitrogen uptake from control plot.

### Economic Analysis

The economic analysis of all the treatments was calculated using the existing market price of input and output in terms of gross returns ($ha^–1^), net returns ($ha^–1^), and the B:C ratio to assess the profitability of different treatments and arrive at an economically viable proposal.

### Statistical Analysis

To test the significance of the results, the conventional strategy provided by Fisher (1992) was used. The critical differences were calculated to measure the importance of differences between treatments. Summary tables with SEM (±) and least significant difference (LSD) (*p* = 0.05) were developed and supplied in the section headed “Results” to illustrate the nature and extent of treatment effects.

## Results

### Growth Attributes

The irrigation level had significant effect on LAI at all the stages of cotton except 30 days where it could not influence LAI (Table 3). LAI improved when irrigation levels raised from 200 to 600 mm. Irrigation level 600 mm recorded the highest LAI at 60, 90, and 120 days and at harvest, which were substantially higher than the LAI observed at 200 mm irrigation level with respective percent increments of 16.15, 16.81, 16.17, and 32.38%.

**TABLE 3:** Effect of irrigation and nitrogen levels on leaf area index of *Bt* cotton.

Treatments	30 DAS	60 DAS	90 DAS	120 DAS	Harvest
**Irrigation levels**					
I_200_	0.170ns	1.313a	1.641a	2.757a	0.281a
I_400_	0.167ns	1.485b	1.857b	3.119b	0.319b
I_600_	0.172ns	1.525b	1.917b	3.203b	0.372c
**Nitrogen levels**					
N_0_	0.152a	1.019a	1.274a	2.140a	0.154a
N_75_	0.166b	1.276b	1.595b	2.680b	0.221b
N_150_	0.178c	1.691c	2.128c	3.552c	0.421c
N_225_	0.183c	1.778c	2.223c	3.734c	0.499d

*Values marked by at least a common letter do not differ significantly according to LSD test (p = 0.05). ns, non-significant.*

The results showed that increasing nitrogen levels from 0 to 225 kg ha^–1^ resulted in a significant enhancement in LAI at all the growth stages, except 30 days where it could not influence LAI (Table 3). Over the remaining nitrogen doses, the largest LAI was obtained with nitrogen level of 225 kg ha^–1^ at 30, 60, 90, and 120 days and at harvest. The percent increment in LAI due to 225 kg N ha^–1^ was 20.39, 74.48, 74.49, 74.49, and 224.03% over control at 30, 60, 90, and 120 days and at harvest, respectively.

The results showed that there was a considerable improvement in DMA at each stage with increasing levels of irrigation (Table 4). The maximum dry matter increased between 60 and 90 days stages. DMA improved with increase in irrigation levels from 200 to 600 mm. Irrigation level 600 mm recorded 16.15, 16.81, 16.17, and 23.73% higher over the irrigation level 200 mm at 60, 90, and 120 days and at harvest, respectively.

**TABLE 4:** Effect of irrigation and nitrogen levels on dry matter accumulation (g plant^–1^) of *Bt* cotton.

Treatments	30 DAS	60 DAS	90 DAS	120 DAS	Harvest
**Irrigation levels**					
I_200_	1.56ns	51.19a	147.70a	220.16a	231.67a
I_400_	1.69ns	57.60ab	166.80b	248.02b	263.36b
I_600_	1.75ns	62.99b	186.06c	284.81c	303.75c
**Nitrogen levels**					
N_0_	1.50ns	44.64a	109.07a	156.48a	166.03a
N_75_	1.58ns	54.42b	144.02b	219.45b	230.37b
N_150_	1.72ns	63.58c	198.34c	294.40c	313.96c
N_225_	1.86ns	66.39c	216.00d	333.66d	354.67d

*Values marked by at least a common letter do not differ significantly according to LSD test (p = 0.05). ns, non-significant.*

The varying levels of nitrogen substantially enhanced the dry matter production of *Bt* cotton at different stages except at 30 days during the experimentation (Table 3). Over the remaining nitrogen doses, the largest DMA was obtained with nitrogen levels of 225 kg ha^–1^ at 30, 60, 90, and 120 days and at harvest. The improvement in DMA due to application of 225 kg N ha^–1^ over control was 24.00, 48.72, 98.04, 113.23, and 113.62% at 30, 60, 90, and 120 days and at harvest, respectively (Table 4).

**FIGURE 2:**
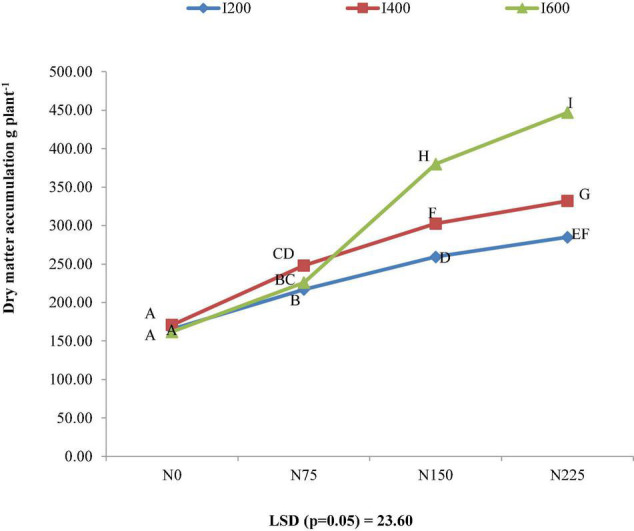
Combined effect of irrigation and nitrogen levels on dry matter accumulation (DMA) at harvest of *Bt* cotton.

It was discovered that the combined influence of irrigation and nitrogen levels on dry matter buildup at harvest was significant. At the same levels of nitrogen, increasing level of irrigation up to 600 mm significantly increases the DMA at harvest. Similarly, at the same level of irrigation, increasing level of nitrogen up to 225 kg ha^–1^ significantly increased the DMA. At harvest, maximum and significant DMA (447.0 g plant^–1^) was recorded in I600 N225 treatment combination, while minimum DMA (165.33 g plant^–1^) was recorded in I200 N0 treatment combination (Figure 2).

Application of 600 mm irrigation level recorded higher CGR over 200 mm irrigation at each growth stage, but found statistically at par with 400 mm irrigation level except at 90–120 days stages where it was significantly superior over both the lower levels of irrigation. Irrigation at 600 mm increased the CGR over 200 mm by 10.34, 23.63, 27.32, 35.95, and 63.63% at 0–30, 30–60, 60–90, 90–120, and 120 days harvest stages, respectively (Table 5).

**TABLE 5:** Effect of irrigation and nitrogen levels on crop growth rate (g m^–2^ day^–1^) of *Bt* cotton.

Treatments	0–30 DAS	30–60 DAS	60–90 DAS	90–120 DAS	120-Harvest
**Irrigation levels**					
I_200_	0.052a	1.65a	3.22a	2.42a	0.33a
I_400_	0.056b	1.86b	3.64b	2.71b	0.44b
I_600_	0.058b	2.04b	4.10b	3.29c	0.54b
**Nitrogen levels**					
N_0_	0.050a	1.44a	2.15a	1.58a	0.27a
N_75_	0.053a	1.76b	2.99b	2.51b	0.31b
N_150_	0.057a	2.06c	4.49c	3.20c	0.56c
N_225_	0.062b	2.15c	4.99d	3.92d	0.60c

*Values marked by at least a common letter do not differ significantly according to LSD test (p = 0.05).*

The graded level of nitrogen had significant influence on CGR during all the growth stages. At 0–30 days stage, the nitrogen @ 225 kg ha^–1^ gave the highest CGR, which was significantly superior over remaining doses. At 30–60 and 120 days harvest stages, the crop growth rate increased significantly up to nitrogen @ 150 kg ha^–1^ over remaining doses of nitrogen. The application of nitrogen @ 225 kg ha^–1^ recorded significantly higher crop growth rate than all the other tested nitrogen rates at 60–90 and 90–120 days stages. The increasing level of nitrogen enhanced the CGR up to the application of N @ 225 kg ha^–1^ and gave 24, 49.30, 132.09, 148.10, and 122.22% higher CGR at all the growth phases over control, respectively (Table 5).

Irrigation levels affected the RGR of *Bt* cotton at each growth stages, except 30–60 days. Irrigation at 600 mm showed significant improvement in RGR at 60–90, 90–120, and 120 days harvest in comparison to irrigation at 400 and 200 mm at each growth stage, except during 60–90 and 90–120 days where it remained at par with irrigation at 400 mm. Irrigation at 600 mm gave 2.84, 3.53, and 3.91% higher RGR at 60–90, 90–120, and 120 days harvest stages over irrigation at 200 mm (Table 6).

**TABLE 6:** Effect of irrigation and nitrogen levels on relative growth rate (g g^–1^ day^–1^) of *Bt* cotton.

Treatments	30–60 DAS	60–90 DAS	90–120 DAS	120-Harvest
**Irrigation levels**				
I_200_	1.70ns	2.11a	2.26a	2.29a
I_400_	1.75ns	2.15b	2.31b	2.34b
I_600_	1.78ns	2.17b	2.34b	2.38c
**Nitrogen levels**				
N_0_	1.64a	1.98a	2.13a	2.16a
N_75_	1.73b	2.10b	2.27b	2.29b
N_150_	1.79c	2.23c	2.39c	2.42c
N_225_	1.80c	2.27d	2.44d	2.47d

*Values marked by at least a common letter do not differ significantly according to LSD test (p = 0.05). ns, non-significant.*

The nitrogen fertilization had progressively and significantly increased the relative growth rate with enhancing dose of nitrogen fertilizer up to 225 kg ha^–1^ at each growth stage. At 30–60, 60–90, 90–120, and 120 days harvest stages, application of nitrogen @ 225 kg ha^–1^ substantially enhanced RGR by 9.75, 14.64, 14.55, and 14.35% over control, respectively (Table 6).

**FIGURE 3:**
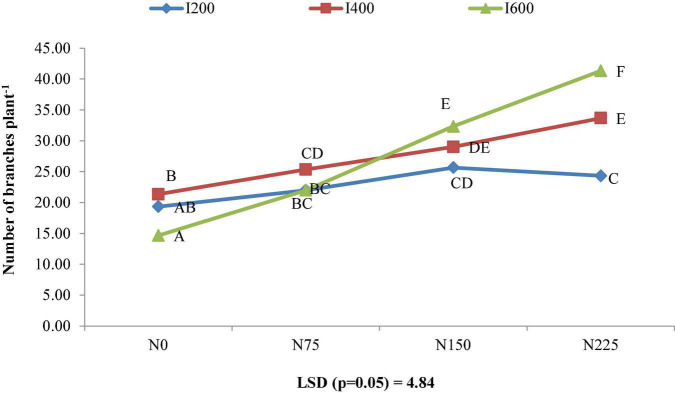
Combined effect of irrigation and nitrogen levels on number of branches plant^–1^ of *Bt* cotton.

The different irrigation levels substantially enhanced the number of branches per plant. The irrigation at 600 mm recorded maximum branches per plant as compared to remaining irrigation levels, but the difference between irrigation level 600 and 400 mm was not significant. An increase of 20.80% in number of branches per plant was recorded under 600 mm level over 200 mm levels of irrigation (Figure 3). The nitrogen application rate 225 kg ha^–1^ enhanced the number of branches plant^–1^ by 14.17, 43.27, and 79.55% in comparison to 150, 75, and 0 kg N ha^–1^, respectively (Figure 3).

### Nitrogen Contents and Uptake

Different irrigation levels progressively increased N content in all the parts (seed, lint, and stalk) of *Bt* cotton. Increasing level of irrigation from 200 to 400 mm substantially enhanced N content in seed and stalk by 11.94 and 8.12%, respectively. Significantly higher nitrogen content in lint of *Bt* cotton was found under irrigation level 600 mm compared to remaining levels of irrigation. Increasing irrigation levels from 200 to 600 mm significantly increased the N uptake by seed, lint, and stalk of cotton crop. The irrigation level 600 mm showed 56.66, 74.05, and 85.72% improvement in nitrogen absorption by seed, lint, and stalk over irrigation level 200 mm and resulted in 69.07% improvement in total N uptake over irrigation level 200 mm (Table 7).

**TABLE 7:** Effect of irrigation and nitrogen levels on N content and uptake of *Bt* cotton.

Treatments	N content (%)			N uptake (kg ha^–1^)			Total N uptake (kg ha^–1^)
	Seed	Lint	Stalk	Seed	Lint	Stalk	
**Irrigation levels**							
I_200_	2.93a	0.31a	0.98a	43.40a	1.85a	31.72a	76.96a
I_400_	3.28b	0.35b	1.05b	61.33b	2.69b	45.16b	109.18b
I_600_	3.42b	0.39c	1.06b	67.99c	3.22c	58.91c	130.12c
**Nitrogen rates**							
N_0_	2.40a	0.19a	0.48a	33.34a	1.07a	15.04a	49.44a
N_75_	3.03b	0.25b	1.06b	48.64b	1.61b	37.66b	87.91b
N_150_	3.65c	0.46c	1.25c	71.07c	3.58c	60.25c	134.90c
N_225_	3.77c	0.49d	1.33d	77.23d	4.09d	68.11d	149.43d

*Values marked by at least a common letter do not differ significantly according to LSD test (p = 0.05).*

**FIGURE 4:**
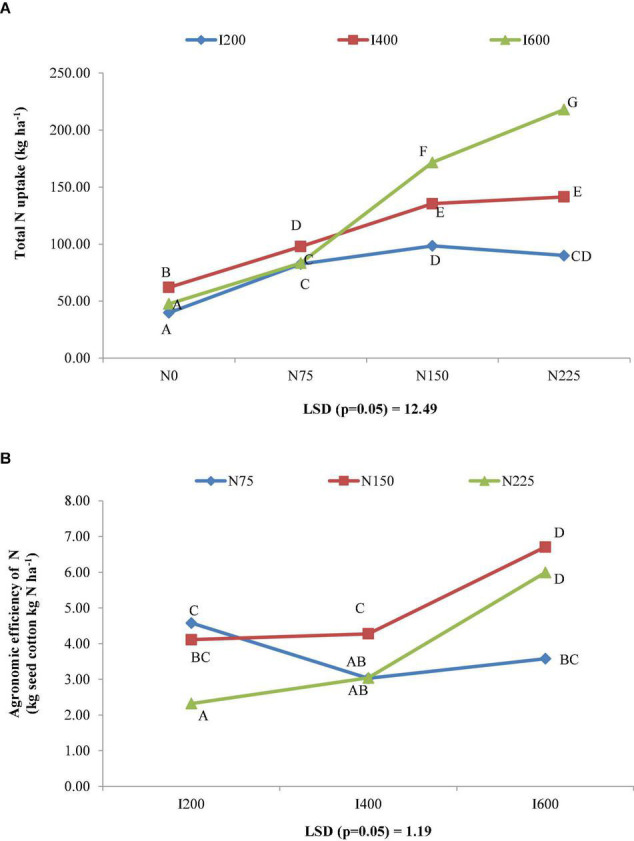
Combined effect of irrigation and nitrogen levels on **(A)** total nitrogen uptake and **(B)** agronomic efficiency of nitrogen (AEN) (kg seed cotton kg N ha^–1^) of *Bt* cotton.

Increasing doses of nitrogen up to 225 kg ha^–1^ significantly improved the N content of lint and stalk. However, seed N content substantially enhanced till 150 kg N ha^–1^. The improvement in N contents of seed, lint, and stalk was 57.08, 157.89, and 177.08%, respectively, over no N application. Similarly, applying 225 kg N ha^–1^ resulted in 131.64, 282.24, 352.85, and 202.64% increment in N absorption by seed, lint, and stalk and total N uptake, respectively, over no N application. Similarly, significantly higher nitrogen uptakes by seed, lint, and stalk along with total absorption by cotton crop were found in nitrogen application @ 225 kg ha^–1^ compared to remaining nitrogen rates. The nitrogen application rate 225 kg ha^–1^ gave 58.77, 154.03, 80.85, and 69.98% and 8.67, 14.24, 13.04, and 10.77% higher nitrogen absorption by all the economic parts and total uptake of *Bt* cotton over 75 and 150 kg ha^–1^, respectively (Table 7). Combined effect of irrigation levels and nitrogen levels on N uptake of *Bt* cotton was found to be significant. The maximum N uptake was observed in I600 N225 treatment combination, while the minimum N uptake was observed in I200 N0 treatment combination (Figure 4).

### Nitrogen Use Efficiency

The different irrigation levels significantly affected various NUE indices such as AEN, REN, PEN, PFPN, and iNUE. The AEN, REN, and PFPN increased with increasing levels of irrigation and the highest values were recorded under 600 mm irrigation. The increment in AEN, REN, and PFPN with irrigation levels 600 mm over 200 mm was 47.95, 76.92, and 23.34%, respectively. The PEN and iNUE decreased with increasing levels of irrigations and irrigation level 200 mm recorded 22.33 and 13.71% higher PEN and iNUE than irrigation level 600 mm (Table 8).

**TABLE 8:** Effect of irrigation and nitrogen levels on nitrogen use efficiency indices of *Bt* cotton.

Treatments	AEN	PEN	REN	PFPN	INUE
**Irrigation levels**					
I_200_	3.67a	9.64a	0.39a	17.59a	29.02a
I_400_	3.45a	7.88a	0.44a	21.21b	25.21b
I_600_	5.43b	7.88a	0.69b	21.69b	25.52b
**Nitrogen levels**					
N_0_	–	–	–	–	–
N_75_	3.73a	7.33a	0.51a	29.88a	25.55a
N_150_	5.03b	9.09b	0.57b	18.11b	20.64b
N_225_	3.79a	8.99b	0.44c	12.50c	20.15b

*Values marked by at least a common letter do not differ significantly according to LSD test (p = 0.05).*

The indices of nitrogen use efficiency such as AEN, REN, and PEN enhanced with increasing levels of N up to 150 kg N ha^–1^ and following that, it decreased. The nitrogen level 150 kg N ha^–1^ gave 34.92, 11.99, and 23.89% higher AEN, REN, and PEN compared to 75 kg N ha^–1^, respectively. The PFPN and iNUE decreased with enhancing levels of nitrogen from 75 to 225 kg ha^–1^ (Table 8).

**FIGURE 5:**
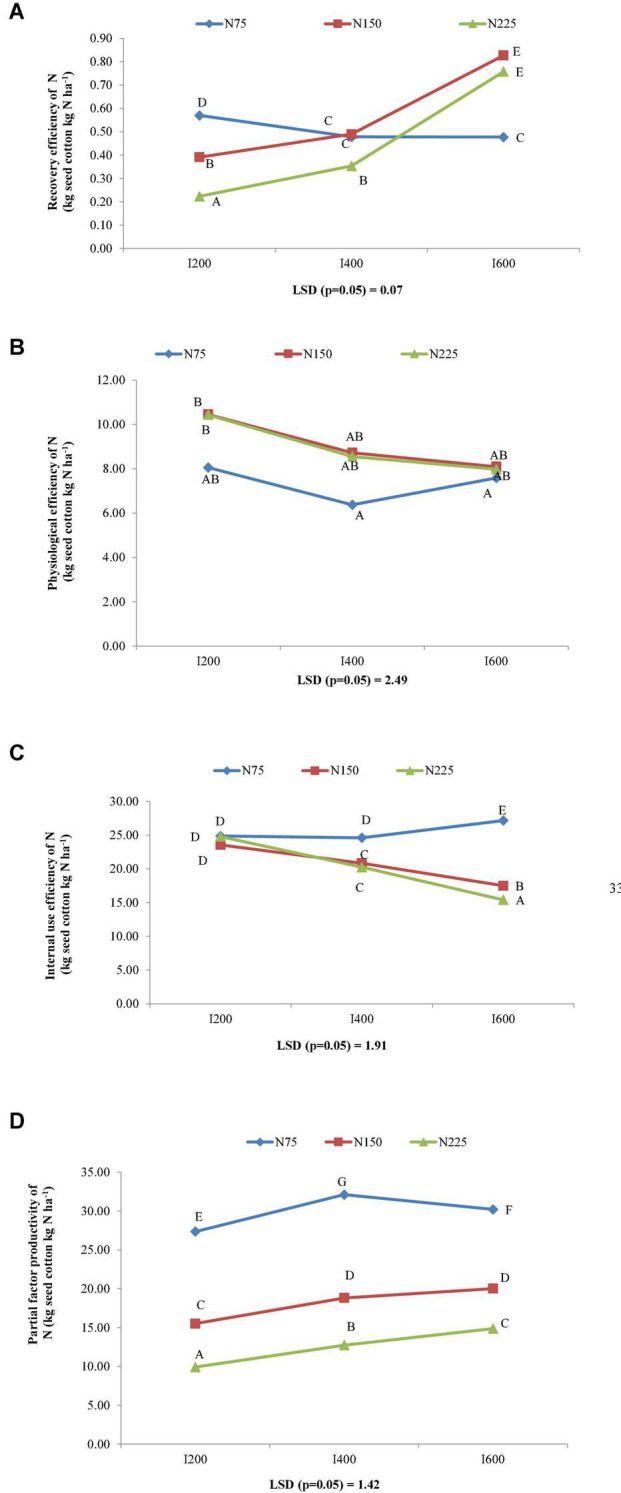
Combined effect of irrigation and nitrogen levels on **(A)** recovery efficiency of nitrogen (REN) (kg seed cotton kg N ha^–1^), **(B)** physiological efficiency of nitrogen (PEN) (kg seed cotton kg N ha^–1^), **(C)** internal nitrogen use efficiency (iNUE) (kg seed cotton kg N ha^–1^), and **(D)** partial factor productivity of nitrogen (PFPN) (kg seed cotton kg N ha^–1^) of *Bt* cotton.

The interactions between irrigation and nitrogen levels were significant for AEN, REN, PEN, PFPN, and iNUE. Application of 600 mm irrigation water with 150 kg N ha^–1^ gave the highest values of AEN (Figure 4B) and REN (Figure 5A) among the treatment combinations. However, these were comparable with application of 600 mm irrigation water with 225 kg N ha^–1^. Significantly superior iNUE (Figure 5C) and PFPN (Figure 5D) were recorded with application of 75 kg N ha^–1^ with 600 and 400 mm irrigation water, respectively, over other treatment combinations. Interestingly, performance of 150 and 225 kg N ha^–1^ at same irrigation level for PEN (Figure 5B) was almost similar with higher values in 150 kg N ha^–1^.

With each rise in irrigation levels, irrigation water productivity of cotton declined significantly from 200 to 600 mm of irrigation. Over 400 and 600 mm irrigation, 200 mm irrigation resulted in 62.5 and 136.36% higher irrigation water productivity, respectively (Table 9).

**TABLE 9:** Effect of irrigation and nitrogen levels on irrigation water productivity of *Bt* cotton.

Nitrogen levels	I_200_	I_400_	I_600_	Mean
N_0_	0.85E	0.55BC	0.33A	0.58a
N_75_	1.03F	0.60C	0.38A	0.67b
N_150_	1.16G	0.71D	0.50B	0.79c
N_225_	1.12G	0.72D	0.56C	0.80d
Mean	1.04c	0.64b	0.44a	

*Values marked by at least a common small letter do not differ significantly for main effects of nitrogen and irrigation treatments according to LSD test (p = 0.05). Values marked by at least a common capital letter do not differ significantly for interaction effect of nitrogen and irrigation treatments according to LSD test (p = 0.05).*

Irrigation water productivity of cotton enhanced significantly as nitrogen levels were raised from 0 to 225 kg ha^–1^, despite the fact that the difference between N 150 and N 225 kg ha^–1^ levels was non-significant. Over the remaining nitrogen rates, the highest irrigation water productivity was found with 225 kg N ha^–1^ (0.80) closely followed by 150 kg N ha^–1^ (0.79) (Table 9).

### Economic Analysis

Increasing irrigation levels progressively improved all the economical parameters of *Bt* cotton. Irrigation level 600 mm resulted in 27.96, 41.50, and 21.34% higher gross return, net returns, and B:C ratio, respectively, compared to irrigation level 200 mm. In comparison to irrigation at 200 mm, irrigation at 400 mm yielded an extra net return of $429 ha^–1^ and a B:C ratio of 0.55 (Table 10).

**TABLE 10:** Effect of irrigation and nitrogen levels on economics of *Bt* cotton.

Cost of cultivation (USD/ha)				
**Nitrogen levels**	**I_200_**	**I_400_**	**I_600_**	**Mean**
N_0_	678	696	714	696
N_75_	702	720	739	720
N_150_	717	735	754	735
N_225_	732	750	769	750
Mean	707	725	744	

**Gross return (USD/ha)**				

***Nitrogen* levels**	**I_200_**	**I_400_**	**I_600_**	**Mean**

N_0_	1557A	1990CD	1821B	1789a
N_75_	1871BC	2196E	2067CDE	2045b
N_150_	2121DE	2577F	2746G	2481c
N_225_	2032D	2613FG	3067H	2570d
**Mean**	1895a	2344b	2425b	

**Net return (USD/ha)**				

**Nitrogen levels**	**I_200_**	**I_400_**	**I_600_**	**Mean**

N_0_	880A	1294CD	1106B	1093a
N_75_	1169BC	1475E	1328D	1324b
N_150_	1404DE	1841F	1992F	1746c
N_225_	1361C	1862F	2298G	1820c
**Mean**	1188a	1617b	1681b	

**B:C ratio**				

**Nitrogen levels**	**I_200_**	**I_400_**	**I_600_**	**Mean**

N_0_	2.30A	2.86CDE	2.55B	2.57a
N_75_	2.67BC	3.05E	2.80CD	2.84b
N_150_	2.96DE	3.50F	3.64F	3.37c
N_225_	2.78CD	3.48F	3.99G	3.42c
**Mean**	2.67a	3.22b	3.24b	

*Values marked by at least a common small letter do not differ significantly for main effects of nitrogen and irrigation treatments according to LSD test (p = 0.05). Values marked by at least a common capital letter do not differ significantly for interaction effect of nitrogen and irrigation treatments according to LSD test (p = 0.05).*

Nitrogen applied up to 225 kg ha^–1^ brought about progressive and significant improvement in gross returns and gave 43.66% higher gross return over no N application. Data indicated that 66.48 and 33.07% increment in net return and B:C ratio were observed when 225 kg N ha^–1^ was applied over control (Table 10).

The interaction between irrigation and nitrogen levels was significant for all the economical parameters (Table 10). Among the treatment combinations, 600 mm irrigation water with 225 kg N ha^–1^ had a considerably higher gross return ($3067 ha^–1^), net return ($2298 ha^–1^), and B:C ratio (3.99).

## Discussion

In this study, appropriate irrigation improved photosynthate accumulation in the aerial region of the cotton plant, which may facilitate assimilate allocation to reproductive organs. The generation of assimilates is linked to the root system [root distribution and physiological activity, as well as the photosystem (light capturing organ, the activity of PSII reactive center, and the utilization or consumption of photochemical energy)]. The root system and leaf photosystem, on the other hand, work together to promote plant growth and development (Kapoor et al., 2020). The enhanced moisture uptake under irrigation at 600 mm combined with nutrients led in increased cell elongation and turgidity, resulting in an increase in LAI and dry matter production (Dadgale et al., 2014), enhanced photosynthesis by allowing the plant to capture a greater amount of solar energy, which increased photosynthate translocation to the developing portions, resulted in higher LAI and DMA (Ahlawat and Gangaiah, 2010; Alse and Jadhav, 2011). The lower amount of irrigation had the lowest LAI and dry matter accumulation because it failed to maintain the ideal soil moisture, which may have inhibited cell elongation, as well as limited photosynthesis and carbohydrate synthesis (Dadgale et al., 2014). In addition, water is the key limiting factor that can regulate cell growth and metabolism, which ultimately affect the crop growth and development, possibly due to this lower irrigation (200 mm) resulted in lower LAI (Farooq et al., 2022) and dry matter accumulation (Farooq et al., 2021). The crop and relative growth rate increased with increasing quantity of dry matter because both of these parameters are highly dependent on crop dry matter. The appropriate soil moisture state, along with adequate irrigation with 600 mm may have contributed to higher nutrient uptake, supporting crop growth and development, as well as a good influence on dry matter, resulting in an increase in cotton crop and relative growth rate (Bhunia, 2007; Ahlawat and Gangaiah, 2010). The better moisture absorption by roots along with nutrients under irrigation level 600 mm could have increase the photosynthesis and enabled the crop to capture a greater amount of radiant energy, which boosted photosynthate translocation to the growth portions, resulting in increased cell elongation and a greater number of branches per plant (Hussein et al., 2011; Dadgale et al., 2014; Mahadevappa et al., 2018; Meena H. et al., 2018).

Nitrogen increases the leaf area and leaf area index (LAI), promotes the synthesis of proteins involved in cell development, cell proliferation, and the development of the cell wall and cytoskeleton (Luo et al., 2020; Sun et al., 2020). The rise in LAI with enhancing nitrogen levels from 0 to 225 kg ha^–1^ could be attributable to nitrogen’s beneficial influence on cotton growth, development, and vegetative components (Gundlur et al., 2013). Increased nitrogen intake resulted in more vegetative growth than reproductive growth. The LAI fell as nitrogen levels were reduced because these treatments failed to fulfill the assimilate needs of the growing sections, resulting in reduced leaf area (Norton and Silvertooth, 2007; Munir et al., 2015).

The favorable effect of nitrogen on cotton growth and development may be explained by the increase in dry matter accumulation with increasing nitrogen levels (Gundlur et al., 2013). According to a previous study, a sufficient supply of nitrogen in the right quantity can promote cell elongation, resulting in greater vegetative growth and more cotton dry matter (Sunitha et al., 2010). However, lower nitrogen levels resulted in lower dry matter accumulation because an insufficient supply of nitrogen from the soil (which was low in available nitrogen) might have inhibited cell elongation, which suppressed plant vegetative growth, resulting in lower dry matter under these conditions as compared to higher nitrogen levels (Reddy et al., 2004; Munir et al., 2015).

Cotton is an indeterminate crop with a lengthy growing season, thus timely nitrogen supply under split application may have increased plant^–1^ DMA, leading to a higher yield and relative growth rate of cotton, which are dependent on plant dry matter accumulation (Giri et al., 2014). The increase in number of branches plant^–1^ might be attributed to nitrogen fertilization because nitrogen is responsible for increasing leaf area index, which enables the plants to produce more branches (Saleem et al., 2010; Ali and Hameed, 2011; Main et al., 2014).

The nitrogen concentration in seed, lint, and stalk, as well as their uptake by these parts, was dramatically affected by irrigation levels. The nitrogen intake of a crop is determined by the plant’s nitrogen concentration and yield. The increase in total nitrogen intake seen in this study is due to a considerable rise in both the content and yield of cotton with increased irrigation (Yadav and Chauhan, 2016). Higher irrigation levels were also associated with higher nitrogen use efficiency indices, which could be because increased water availability increased nitrogen availability through enhanced mineralization, as well as improved mobility of nitrogen to the root surface, resulting in an increased uptake and concentration in plants (Saini, 2017).

Cotton’s nitrogen content and absorption improved as nitrogen fertilization increased. With N 225 kg ha^–1^, the highest nitrogen content in lint was discovered, as well as its uptake by all the plant components individually and the plant as a whole. Higher nitrogen application rates to the soil may have increased nitrogen availability to *Bt* cotton plants, resulting in increased LAI development and ultimately photosynthates generation. As a result of the increased demand for nitrogen, more nitrogen was taken up from the soil and accumulated in various plant parts, resulting in higher nitrogen concentrations in plant sections. Higher nitrogen intake was the result of higher yield combined with higher nitrogen content in plant sections. Increased seed index and seed production, as well as a higher level of nitrogen fertilization, have resulted in a higher N concentration in seed (Boquet and Breitenbeck, 2000; Bhalerao et al., 2011).

The nitrogen use efficiency (AEN, PEN, and REN) improved until it reached 150 kg N ha^–1^, then dropped as nitrogen application increased. This could be attributed to the fact that increasing nitrogen doses beyond 150 kg ha^–1^ failed to result in a proportionate rise in seed cotton production. Cotton had the highest nitrogen use efficiency when nitrogen was administered at 150 kg ha^–1^ because increased nitrogen levels resulted in a balanced increase in vegetative and reproductive components, resulting in better seed cotton yields (Fritschi et al., 2003). High nitrogen application (225 kg ha^–1^), on the other hand, can shift the balance in favor of vegetative growth, delaying crop maturity, and diminishing cotton yield. The decreased AEN with greater doses of N was attributed to the fact that the rise in seed cotton production was not proportional to the N application rate and uptake (Aujla et al., 2005; Gangaiah et al., 2013; Yadav and Chauhan, 2016).

With an increase in irrigation levels, the water productivity gradually declined. Water productivity was highest when 200 mm irrigation was used, followed by 400 and 600 mm irrigation levels. The impact of irrigation levels on water productivity differs from the influence on yield, which grew as irrigation levels climbed. The decrease in water productivity seen in this study is explained by the significantly smaller gain in yield compared to the amount of irrigation administered with an increase in irrigation levels (Nalayini et al., 2006; Pawar et al., 2013).

It was observed that water productivity enhanced as nitrogen rates increased. Increased leaf area leads to a reduction in the evaporation component of evapotranspiration, a lesser increase in evapotranspiration compared to yield and better usage of available soil water, as demonstrated in this study. The findings are in line with previous study, which showed that adding nitrogen to a nitrogen-deficient soil boosted water productivity when water was available (Yadav and Chauhan, 2016).

Irrigation enhanced the net return and B:C ratio substantially. In comparison to irrigation at 200 mm, irrigation at 600 mm yielded an additional net return of $493 ha^–1^ and B:C ratio of 0.57. This rise was mostly owing to a comparatively higher economic yield with a lower added cost under this treatment, which resulted in higher returns.

Irrigation and nitrogen levels influenced dry matter aggregation at harvest, number of branches plant^–1^, total nitrogen intake, nitrogen use efficiency, irrigation water productivity, and economics of *Bt* cotton. In compared to all the other factors, in general, 600 mm irrigation and 225 kg ha^–1^ nitrogen rate greatly enhanced these metrics. These results can be explained by an undersea shortage, which indicated that the N was not assimilated at the rate required for optimal growth. Despite the fact that it was administered, it was hypothesized that N was lost due to volatilization. Furthermore, due to the silty clayey soils, some nitrogen was allegedly lost through leaching. Decreased N dosages sprayed in coverage lead to decreased productivity, regardless of irrigation level (Aujla et al., 2005). The amount of water in the soil under optimal conditions is critical for plant N uptake. To avoid waste of inputs, proper water management is required. Cotton yield response to increased irrigation levels was linear for all the N levels employed in the water treatments (Singh et al., 2010).

## Conclusion

Irrigation and nitrogen management are the two most important factors for improving the growth and yield of cotton. In this study, significantly higher growth attributes and net return and benefit cost ratio were found with irrigation level 600 mm and nitrogen level 225 kg ha^–1^ in the hot arid region of Rajasthan that could be ascribed to the ideal soil moisture content for better nutrient absorption by plants. However, applying a reduced rate of nitrogen (150 kg N ha^–1^) improved the NUE indices such as agronomic efficiency and recovery efficiency of N fertilizer at 600 mm irrigation over 225 kg N ha^–1^ at the same irrigation level. However, its performance in terms of growth attributes and profitability indices such as net return and B:C ratio was inferior to irrigation 600 mm with 150 kg N ha^–1^. So, application of 600 mm of irrigation along with 225 kg N ha^–1^ could be recommended for achieving higher growth and yield, as well as profitability of *Bt* cotton under hot arid region of Rajasthan and similar agroecologies.

## Data Availability Statement

The raw data supporting the conclusions of this article will be made available by the authors, without undue reservation.

## Author Contributions

All authors contributed significantly in conceptualization, writing, editing, and review of the current manuscript and agreed to the submission of the current manuscript.

## Conflict of Interest

The authors declare that the research was conducted in the absence of any commercial or financial relationships that could be construed as a potential conflict of interest.

## Publisher’s Note

All claims expressed in this article are solely those of the authors and do not necessarily represent those of their affiliated organizations, or those of the publisher, the editors and the reviewers. Any product that may be evaluated in this article, or claim that may be made by its manufacturer, is not guaranteed or endorsed by the publisher.
